# Supplementary material for: Doughnut of social and planetary boundaries monitors a world out of balance
Source: Nature. 2025 Oct 1;646(8083):47–56. doi: 10.1038/s41586-025-09385-1 (PMC12488500; doi:10.1038/s41586-025-09385-1)
Supplement: Supplementary file 1 — Supplementary Discussions 1 and 2 and Supplementary Tables 1 and 2 [file 41586_2025_9385_MOESM1_ESM.pdf]

nature portfolio

https://doi.org/10.1038/s41586-025-09385-1

Supplementary information

Doughnut of social and planetary boundaries monitors a world out of balance

In the format provided by the authors and unedited

Doughnut of social and planetary boundaries monitors a world out of balance

Andrew L. Fanning and Kate Raworth

SUPPLEMENTARY INFORMATION

Summary

This Supplementary Information document describes how we selected social indicators and collected data to track shortfall within each dimension of the social foundation, and similarly, how we selected ecological indicators and collected data to track overshoot within each dimension of the ecological ceiling. Supplementary Tables referenced in the Main text are also provided.

Supplementary Discussion 1 – The social foundation: dimensions and indicators

We retain a total of twelve social dimensions, as in the 2017 Doughnut, but we revise three of them to enhance the conceptual clarity of the social foundation compared to the previous version (connectivity, social cohesion, and equality). Connectivity focuses on access to essential infrastructure that connects people to their communities, both physically and digitally. It replaces the 'networks' dimension in the 2017 Doughnut, which combined infrastructural networks with social-support networks: here we propose that such social connectedness is better reflected within a new 'social cohesion' dimension. Social cohesion focuses on the depth of inequalities within a society, i.e. people's sense of belonging within a community, and on the strength of relationship between its members[1]. It replaces the 'social equity' dimension in the previous version, which focused on the relative incomes of its members. The newly named 'equality' dimension focuses on the breadth of inequalities within a society, i.e. social, economic, and political inclusion of all people, irrespective of intersecting differences in identity by gender, race, or other status (as per Goal 10.2 of the SDGs[2]). In doing so, it broadens the scope of the 'gender equality' dimension included in the 2017 version of the Doughnut.

Within the social dimensions, we add two additional indicators with global coverage that have become available since the previous update (food security and access to public transport). We also include a third indicator - racial equality - that currently lacks available data, but we agree with human rights scholars that this deficiency is important to make visible and redress so that global monitoring better reflects the explicit racial- and ethnic-justice commitments of the international community[3,4]. Furthermore, we replace three indicators with alternative proxies that we believe better reflect broader concerns within their respective dimensions (coverage of essential health services, country-specific societal poverty lines set at least $15 a day, and governance by autocratic regimes; previously life expectancy, a single international poverty line set at $3.10 a day, and the Voice and Accountability Index, respectively).

Values reported in the Main text are population-based aggregates of large samples of national data across all countries (World), and across the subset of countries within each of the three income-based country clusters (poorest 40% of countries, middle 40% of countries, and richest 20% of countries). Throughout the analysis, we use national population data collected from *World Population Prospects 2022*[5] and Gross National Income (GNI) per capita collected from *Human Development Report 2023-2024*[6]. The following sub-sections describe the revised set of dimensions and indicators included in the Doughnut's social foundation, and Supplementary Table 1 provides an overview of the 22 indicators

included in our global and country-cluster analyses, including country coverage, descriptions, and data sources.

Food
All people need to have secure access to sufficient, affordable, safe and nutritious food on a daily basis. Ending hunger and achieving food security is the focus of SDG Goal 2. We include two indicators from the SDG Indicators database to track food shortfalls in the Doughnut: (i) the prevalence of undernourishment (SDG 2.1.1[7]), and (ii) the prevalence of moderate to severe food insecurity (SDG 2.1.2[8]). We note that the undernourishment indicator is estimated more quantitatively from national food supply tables, while the food insecurity indicator is measured more qualitatively based on household survey responses. Although both indicators aim to estimate how many people are not getting enough to eat, they are not strictly comparable, and they both have limitations discussed below. Given the growing recognition of land tenure in ensuring household food security and nutrition, especially among rural people[10], we also explored an internationally comparable indicator of land tenure security (SDG 1.4.2[11]) but it is not yet available globally.

Undernourishment is defined as the condition by which a person lacks regular access to an amount of food that is sufficient to provide the energy required for conducting a normal, healthy and active life, given their own dietary energy requirements[7]. This indicator is a widely used source of comparable global information on the energy requirements available for a population to avoid hunger over time (i.e. calories in the food supply), but its focus on population-wide caloric intake does not measure dietary diversity or nutritional status, nor is it well-suited to assess inequalities in food consumption within countries[12]. This indicator was also included in the 2017 Doughnut and national data were collected from the SDG Indicators database. Countries with fewer than 10 observations in total were excluded, missing values were interpolated linearly to yield a balanced panel of 166 countries from 2000–2022, and population-weighted averages were calculated for the full sample and the subset of countries within each country cluster.

Moderate levels of food insecurity are associated with various forms of diet-related health conditions, such as micronutrient deficiency and unbalanced diets, while severe levels of food insecurity imply a high probability of more severe forms of undernutrition, including hunger[8]. This indicator is measured on the self-reported Food Insecurity Experience Scale (FIES), a measurement standard established relatively recently by the UN Food & Agriculture Organisation that consists of eight questions regarding people's access to sufficient food[13]. Although the FIES combines subjective and objective questions, which can help make visible deprivations that cannot otherwise be monitored, we note that scholars have found that this self-reported metric may suggest a high prevalence of FIES among populations unlikely to be undernourished, based on other measures[14]. These authors recommend the FIES should be included alongside other more objective measures of undernourishment, which we follow here (although the undernourishment indicator also has limitations, as noted above). This indicator is a new addition compared to the 2017 Doughnut and national data were collected from the SDG Indicators database. Countries with fewer than 5 observations in the relatively recent dataset were excluded, missing values were interpolated linearly to yield a balanced panel of 126 countries from 2015–2022, and population-weighted averages were calculated for the full sample and for the subset of countries within each country cluster.

We note a seemingly paradoxical divergence between the two food indicator trends reported in Table 1 and Figure 2, with food insecurity increasing and undernourishment declining (+1.1 %pt and -0.2 %pt per year, respectively). We believe these diverging trends result from two main factors. First, the food insecurity time series is considerably shorter than the undernourishment series (2015–2022 and 2000–2022, respectively). Both indicators show a rise in global hunger and food insecurity in the wake of the COVID-19 pandemic, but we can expect the trend effect of this increase on the 8-year food insecurity series will be larger than the same effect on the 23-year undernourishment series. To test this

hypothesis, we estimated the undernourishment trend over the comparable 2015–2022 period and, as expected, undernourishment also shows a statistically significant worsening trend over this shorter period (+0.3 %pt per year). Second, this raises the question of why the undernourishment trend is increasing more slowly than the food insecurity trend. Although the indicators are not strictly comparable, we believe a likely reason is due to our inclusion of 'moderate' food insecurity as being in shortfall, which reflects a higher standard than the approach used to define undernourishment (which has been shown to be very strongly correlated with 'severe' food insecurity[9]).

Health

Universal access to health services reduces the prevalence of disease and provides all people with the essential care for illness and injury that they need from birth to death. Ensuring healthy lives and promoting wellbeing for all is the focus of SDG Goal 3. We include two indicators to track global health shortfalls in the Doughnut: (i) population living in countries with under-five mortality rate exceeding 25 per 1,000 live births (SDG 3.2.1[15]), and (ii) population living in countries lacking high coverage of essential health services (Universal Health Coverage Index score of less than 60) (SDG 3.8.1[16]).

The under-five mortality rate is defined as the probability of a child dying before the age of five years, subject to age-specific mortality rates during their year of birth, expressed as deaths per 1,000 live births[15]. Mortality rates among young children are a central outcome indicator for child health and an established proxy indicator for broader social and economic concerns. This indicator was also included in the 2017 global Doughnut, and we use the same international target to reduce under-five mortality to at least as low as 25 per 1,000 live births (as identified in SDG Target 3.2).

National data were collected from the SDG Indicators database. Countries with fewer than 10 observations in total were excluded, missing values were interpolated linearly to yield a balanced panel of 191 countries from 2000–2022, and the population living in countries exceeding the 25 per 1,000 target was summed for the full sample and for the subset of countries within each country cluster. We note that this approach reports the share of global population in deprivation on the basis of country-level outcomes (rather than individual-level outcomes), which is sensitive to countries with large populations that cross the target. For example, we observe a large improvement in this indicator in 2005 due to China achieving the minimum social standard for under-five mortality in this year (see Extended Data Figure 5).

Coverage of essential health services is the focus of SDG Target 3.8, which spans multiple health domains, including health protection, promotion, prevention, treatment and care[16]. Given the multi-faceted nature of essential health services, the World Health Organisation has constructed the Universal Health Coverage (UHC) service coverage index on a 0-100 scale, based on the geometric mean of 14 normalised proxy indicators organised by four broad categories of service coverage: (i) reproductive, maternal, newborn, and child health, (ii) infectious diseases, (iii) non-communicable diseases, and (iv) service capacity and access (SDG 3.8.1[16]). This indicator replaces the life expectancy measure included in the 2017 Doughnut, and we use an international target to achieve at least 'high' coverage (60 out of 100 or more), as defined by the WHO and World Bank's *Tracking universal health coverage: 2023 global monitoring report*[17].

National data were collected from the SDG Indicators database. Countries with fewer than five observations in the relatively sparse dataset were excluded, missing values were interpolated linearly to yield a balanced panel of 190 countries from 2000–2022, and the population living in countries lacking 'high' UHC Index coverage (less than 60) was summed for the full sample and for the subset of countries within each country cluster. We note that this approach reports the share of global population in deprivation on the basis of country-level outcomes (rather than individual-level outcomes), which is sensitive to countries with large populations that cross the target. For example, we observe large improvements in this indicator in 2007 and 2017 due to China and India achieving the minimum social standard for health service coverage in each of these years, respectively (see Extended Data Figure 5).

We note that the UHC index can be considered an indication of the *average* coverage of essential services within a country, but this is not the same as the percentage of the population who are *fully* covered by a set of essential services[17]. National estimates of the population not covered by essential health services are not currently available however, although there is a global estimate from Ref[17], which finds nearly 60% of humanity was not fully covered by essential health services in 2021 – twice as large as our result tracking the population living in countries without 'high' coverage in the same year. According to the World Health Organisation[17], the agency responsible for the UHC service coverage monitoring framework of SDG 3.8.1, the methodology is being updated to better reflect effective coverage and unmet needs in their next monitoring report, due in 2025. The Doughnut will incorporate such improvements as they become available in future updates.

Education
Education is a fundamental part of every person's lifelong ability to participate in society with dignity. Ensuring quality education and lifelong learning opportunities for all is the focus of SDG Goal 4. We include two indicators to track global education shortfalls in the Doughnut: (i) prevalence of illiteracy among adults aged 15+ (UNESCO[18]), and (ii) proportion of young adults aged 21-23 who have not completed secondary school(SDG 4.1.2[19]).

Achieving literacy and numeracy for youth and adults is the focus of SDG Target 4.6, but the official SDG indicator that tracks the proportion of youth and adults who have achieved or exceeded a fixed level of proficiency (SDG 4.6.1[20]) is not yet available for a large number of countries over our analysis period. Instead, we include the related adult literacy rate, defined by UNESCO as the percentage of people aged 15 and above who can both read and write with understanding a simple statement about their everyday life[18]. This indicator was also included in the 2017 Doughnut and national data were collected from the World Development Indicators database[21]. Countries with fewer than 5 observations in the relatively sparse dataset were excluded, missing values were interpolated linearly to yield a balanced panel of 127 countries from 2000–2022, and population-weighted averages were calculated for the full sample and the subset of countries within each country cluster.

Ensuring that all boys and girls complete primary and secondary education is the focus of SDG Target 4.1. We include the upper secondary completion rate, defined as the percentage of a cohort of young people aged 3-5 years above the intended completion age of 18 years, who have actually completed the upper secondary level[19]. The well-known benefits of completing subject- and skill-oriented instruction during secondary education include preparing young people for tertiary education or the workforce, reducing risks of anti-social activities such as juvenile delinquency and drug abuse, and lowering fertility rates and teenage pregnancies, among others[22]. This indicator replaces the lower secondary enrolment indicator included in the 2017 Doughnut and national data were collected from the SDG Indicators database. Countries with fewer than 5 observations in the relatively sparse dataset were excluded, missing values were interpolated linearly to yield a balanced panel of 116 countries from 2000–2022, and population-weighted averages were calculated for the full sample and the subset of countries within each country cluster.

Income & work
Sufficient income and meaningful work that is safe and fairly paid are essential for households to meet many of their needs and wants with dignity. Ending poverty in all its forms, including income poverty, is the focus of SDG Goal 1 and promoting decent work for all is part of the commitments in SDG Goal 8. We include two indicators to track global income and work shortfalls in the Doughnut: (i) population living below the societal poverty line, set at half their country's median household income or at least $15 a day (adapted from World Bank[23]), and (ii) proportion of young people aged 15-24 not in employment, education, or training (SDG 8.6.1[24]). These indicators are far from ideal proxies for the broader concerns of ensuring sufficient income and decent work for all, but we believe they are better than other options currently available, as discussed below.

Eradicating extreme poverty for all people everywhere is the focus of SDG Target 1.1. However, the extreme international poverty line of $2.15-day (in 2017 PPP prices) – or $785 per year – included in SDG Indicator 1.1.1 is unacceptably low for the purposes of setting a minimum standard for the social foundation of the Doughnut. Ideally, we would include an estimate of poverty that directly measures the 'cost of basic needs' provisioning in a comparable framework across countries (as per Recommendation 15 of the Commission on Global Poverty[25]), but such a metric does not yet exist at a decent minimum standard, as far as we are aware[26,27]. In the interim, we have included an adapted version of the societal poverty line, a relatively new indicator of income poverty that was introduced by the World Bank in 2018[23] that combines absolute and relative concepts of income poverty (as per Recommendation 16 of the Commission on Global Poverty[25]).

Societal poverty is defined by the World Bank as a combination of extreme poverty, which is an absolute floor for everyone, and a relative component that differs across countries as median household consumption (or income) increases above the absolute floor[28]. We calculated societal poverty headcounts by adapting the World Bank method[23,28]; raising the absolute floor to $15-day (rather than the extreme international poverty line of $2.15-day used as an absolute floor by the World Bank). A country's societal poverty headcount is selected as the larger value of either: (a) the number of people living on less than 50% of their country's median household income, or (b) the number of people living on less than $15-day.

A threshold of $15-day – or $5,475 per year – was selected as the minimum standard based on empirical observations suggesting that this threshold is generally associated with a permanent escape from poverty in longitudinal studies of Brazil, Mexico, Chile, and Indonesia[29]. However, we do not know whether the same holds true in other countries that may have different national poverty lines and different price levels for essential goods, which are structural weaknesses of using broad-gauge purchasing power to measure poverty in the World Bank's International Poverty Line[27]. That said, the $15-day line is broadly equivalent to median levels of household income per person currently observed in upper middle-income countries that achieve fairly decent social outcomes, such as Costa Rica, Poland, and Thailand[30], which makes it a conservative minimum standard for this interim indicator, in our view.

This indicator replaces the indicator of population living on less than the $3.10-day international poverty line included in the 2017 Doughnut, and we calculated societal poverty headcounts using national household income data from the World Bank's *Poverty and Inequality Platform*[31]. Countries without observations before 2000 or after 2012 were excluded, and missing values were interpolated linearly to yield a balanced panel of 112 countries from 2000–2022. A set of 19 wealthy nations, such as Australia, the United States, and most of Western Europe, are the only ones in which 50% of regional median household income per person is larger than $15 per day. We set poverty lines for these countries at half of their respective median household income values (averaged over the analysis period), which ranged from $20–35 per day, and retrieved headcounts for these country-specific poverty lines from the *Poverty and Inequality Platform*. Population-weighted averages of population living below country-specific societal poverty lines were then calculated for the full sample and the subset of countries within each country cluster for each year.

Achieving full employment and decent work for all women and men is the focus of SDG Target 8.5. Ideally, a composite indicator of decent working conditions across countries would measure progress towards achieving this target, but no such metric is available globally despite decades of debate, arguably because measuring the quality of employment is inherently normative and contested - especially between workers' and employers' groups[32]. As a proxy for the availability of work, we include the proportion of young people aged 15-24 not in education, employment, or training (youth NEET rate). This indicator replaces the youth unemployment rate indicator included in the 2017 Doughnut, and national data were collected from the SDG Indicators database. Countries with fewer than 5

observations in the relatively sparse dataset were excluded, missing values were interpolated linearly to yield a balanced panel of 119 countries from 2005–2022, and population-weighted averages were calculated for the full sample and the subset of countries within each country cluster.

We note that the youth NEET rate serves as a broader measure of employment possibilities than the unemployment rate due to the inclusion of young discouraged workers as well as those who are outside the labour force due to disability and household chores, while accounting for those young people who are not in the labour force due to education[24]. However, again, it does not account for the quality of employment, thus it likely undercounts young people who must accept low-paid and/or exploitative work to meet their needs.

Water & sanitation
Clean water is a daily essential for drinking, bathing, cooking, washing clothes, and avoiding the spread of water-borne diseases like cholera. Ensuring safely managed drinking water and sanitation for all is the focus of SDG Goal 6. We include two widely used indicators to track global water and sanitation shortfalls in the Doughnut: (i) population lacking access to safely managed drinking water (SDG 6.1.1[33]), and (ii) population lacking access to safely managed sanitation services (SDG 6.2.1[34]).

Achieving universal and equitable access to safe and affordable drinking water is the focus of SDG Target 6.1. Safely managed drinking water services are defined as an improved drinking water source which is accessible on premises, available when needed, and free from contamination[33]. 'Improved' drinking water sources include piped supplies, boreholes and tubewells, protected dug wells, protected springs, rainwater, water kiosks, and packaged and delivered water. This indicator updates the drinking water indicator in the 2017 Doughnut, which measured access to 'improved' drinking water sources but did not explicitly monitor accessibility or cleanliness, and national data were collected from the SDG Indicators database. Countries with fewer than 10 observations in the dataset were excluded, missing values were interpolated linearly to yield a balanced panel of 124 countries from 2000–2022, and population-weighted averages were calculated for the full sample and the subset of countries within each country cluster. We note that this indicator currently only covers countries with 50-55% of the global population (the lowest share across all the social indicators included in our analysis), and is missing national estimates for several large countries, such as China and India, among others, which will be incorporated into future updates of the Doughnut as this data becomes available. In addition, it would be desirable to track the affordability of water provisioning services, ideally by private versus public management regimes, but such a metric is not currently available globally.

Achieving access to adequate and sustainable sanitation and hygiene for all is the focus of SDG Target 6.2. Safely managed sanitation services are defined as an improved sanitation facility that is not shared with other households and where excreta are safely disposed of in situ, or removed and treated off-site[34]. 'Improved' sanitation facilities include flush and pour flush toilets connected to sewers and septic tanks and dry sanitation technologies such as dry pit latrines with slabs, ventilated improved pit latrines and composting toilets. This indicator updates the sanitation indicator in the 2017 Doughnut, which measured access to 'improved' sanitation services irrespective of whether the facilities were shared and did not explicitly monitor the safe disposal of excreta. National data were collected from the SDG Indicators database. Countries with fewer than 10 observations in the dataset were excluded, missing values were interpolated linearly to yield a balanced panel of 131 countries from 2000–2022, and population-weighted averages were calculated for the full sample and the subset of countries within each country cluster.

Energy
Gaining access to reliable electricity can be transformative for education, household life, community services, and the local economy. In addition, removing dependencies on dirty fuels – like wood, dung, and kerosene for cooking – avoids indoor air pollution, especially affecting women and girls. Ensuring

access to affordable, reliable, and sustainable energy for all is the focus of SDG Goal 7. We include two widely used indicators to track global energy shortfalls in the Doughnut: (i) population lacking access to electricity (SDG 7.1.1[35]), and (ii) population lacking access to clean fuels and technologies for cooking, heating and lighting (SDG 7.1.2[36]).

Ensuring universal access to affordable and reliable energy services is the focus of SDG Target 7.1. Access to electricity is defined as the presence of an electricity connection in the household that serves as the primary source of lighting from the local electricity provider, solar systems, mini-grids, or stand-alone systems[35]. It would be highly desirable to monitor additional key energy attributes beyond 'connected or not', such as capacity, reliability, affordability, and safety. While such metrics are beginning to be developed[37–40], they are not yet available globally from international databases. This indicator was included in the 2017 Doughnut, and national data were collected from the SDG Indicators database. A balanced panel of 193 countries was collected over 2000–2022, and population-weighted averages were calculated for the full sample and the subset of countries within each country cluster.

Access to clean fuels for cooking, heating, and lighting is defined by evidence-based guidance from the World Health Organisation for indoor air quality and household fuel combustion[41], where 'clean' is assessed with respect to emission-rate targets and specific recommendations to avoid unprocessed coal and kerosene in the home[36]. This indicator updates the indicator on inadequate cooking facilities in the 2017 Doughnut, which did not measure other energy end-uses in the home or fuel emission rates that may vary depending on technologies available, and national data were collected from the SDG Indicators database. Countries with fewer than 10 observations in the dataset were excluded, missing values were interpolated linearly to yield a balanced panel of 187 countries from 2000–2022, and population-weighted averages were calculated for the full sample and the subset of countries within each country cluster.

Connectivity
People need to be able to connect with others: mobility services and digital communications are both important means of generating opportunities in life, connecting people to essential services, employment and community. Providing safe, affordable, accessible, and sustainable transport systems, notably by expanding public transport, is the focus of SDG Target 11.2, while increasing access and enhancing the use of information and communications technology are the foci of SDG Target 9.c and SDG Target 17.8. We include two indicators to track global connectivity shortfalls in the Doughnut: (i) urban population lacking convenient access to public transport (SDG 11.2.1[42]), and (ii) population not using the Internet (SDG 17.8.1[43]). We note that 'connectivity' is a new dimension in the Doughnut that replaces the previous 'networks' dimension, as described in the introduction to this social foundation section.

Access to public transport in urban areas is considered convenient when a stop is accessible either within a 500 m walking distance along the street network from a reference point, such as a home, school, work place, market, etc. to a low-capacity public transport system (e.g. bus, Bus Rapid Transit), or within 1 km to a high-capacity system (e.g. rail, metro, ferry)[42]. This is an important new global mobility indicator, only available for 2020 at the time of writing, but with clear intention to monitor on an ongoing basis. It is a new addition to the Doughnut compared to the 2017 version, and national data were collected from the SDG Indicators database. There were 177 countries with available data for this indicator in 2020, and population-weighted averages were calculated for the full sample and the subset of countries within each country cluster.

Access to the Internet has changed the way most people live, work, and communicate in the 21st century. The proportion of people using the Internet is defined as the proportion of individuals who used the Internet from any location in the last three months, and highlights the importance of Internet use as an enabler and helps to measure the digital divide, which, if not properly addressed, will aggravate inequalities in all social domains[43]. This indicator updates the Internet access indicator in the 2017 Doughnut, which did not consider Internet users in locations outside the home, and national data were

collected from the SDG Indicators database. Countries with fewer than 10 observations in the dataset were excluded, missing values were interpolated linearly to yield a balanced panel of 190 countries from 2000–2022, and population-weighted averages were calculated for the full sample and the subset of countries within each country cluster.

Housing
People living in sustainable and resilient homes have better health, improved capacity to create thriving communities, and are less vulnerable to the risks of natural hazards and climate change. Ensuring access to adequate, safe, and affordable housing for all is the focus of SDG Target 11.1. We include one indicator to track global housing shortfalls in the Doughnut: proportion of urban population living in slums, or informal settlements (SDG 11.1.1[44]). This indicator updates the housing indicator in the 2017 Doughnut, which only monitored urban populations in global South countries, and national data were collected from the SDG Indicators database. Countries with fewer than 5 observations in the relatively sparse dataset were excluded, missing values were interpolated linearly to yield a balanced panel of 170 countries from 2000–2022, and population-weighted averages were calculated for the full sample and the subset of countries within each country cluster.

It would be desirable to monitor additional aspects of housing, such as the quality of housing in rural areas or the extent of homelessness, but such metrics are not currently available globally. In addition, we note that there are relatively few global North countries included in this dataset, despite the ambition of adequate housing for all articulated in SDG 11.1.

Population living in slums or informal settlements are defined according to minimum standards across the following four criteria: (i) access to improved water and sanitation, (ii) sufficient living area, (iii) structural quality, durability, and location, and (iv) security of tenure[44]. Although there is some overlap in these criteria with the indicators assessing shortfalls in access to safely managed water and sanitation, it is not currently possible to disaggregate the data by criterion. We note the SDG Indicator database includes an additional series that monitors SDG 11.1.1 in terms of ‘inadequate housing’, which is defined according to the four criteria listed above as well as a fifth ‘affordability’ criterion. This metric would be preferable for our purposes of defining minimum acceptable social standards, but it is currently only available for a relatively small number of 30-40 wealthy countries.

Equality
Every person should have an equal opportunity to make the most of their life. This is regardless of differences of identity, such as gender and race, while recognising that historically groups of people have experienced significant discrimination based on these characteristics. Empowering and promoting the social, economic, and political inclusion of all, irrespective of age, sex, disability, race, ethnicity, religion or other status is the focus of SDG Target 10.2, and SDG Goal 5 is wholly focused on achieving gender equality and empowering all women and girls. We include space for two indicators to track global equality shortfalls in the Doughnut: (i) Gender Inequality Index, which tracks the gap between women and men in terms of reproductive health, empowerment, and employment (UNDP[6]), and (ii) a ‘missing data’ space for an indicator yet to be defined that monitors progress towards achieving the racial and ethnic equality commitments of the international community, including the 2030 Agenda. We note that this is a new dimension in the Doughnut that replaces the previous ‘gender equality’ dimension in the 2017 version, as described in the introduction to this social foundation section.

The Gender Inequality Index is a core component of the UNDP’s flagship *Human Development Report* that annually benchmarks the current state and evolution of gender parity across three key dimensions (reproductive health, empowerment, and the labour market)[6]. It provides a proxy composite indicator of progress across many of the targets identified in SDG Goal 5. Scores can be interpreted as the percentage of the gender gap remaining. This indicator merges and updates the two political and economic gender equality indicators in the 2017 Doughnut, which measured the proportion of seats held by women in national parliaments and the gender pay gap, respectively, and national data were

collected from UNDP's *Human Development Report 2023-2024*[6]. Countries with fewer than 10 observations in the dataset were excluded, missing values were interpolated linearly to yield a balanced panel of 155 countries from 2000–2022, and population-weighted averages were calculated for the full sample and the subset of countries within each country cluster.

We have been struck by the lack of targets and metrics required to track the explicit racial- and ethnic-equality commitments of the 2030 Agenda. This point has likewise been noted by a UN Human Rights Council Special Rapporteur report that finds racial- and ethnic-justice commitments are largely absent from the operationalization of the SDGs, as seen through the lack of disaggregation in the SDG Targets and Indicators by race or ethnicity[3]. For example, SDG Target 10.2 is the only target that explicitly mentions racial and ethnic equality but the only indicator for this target, SDG 10.2.1[45], removes the aspirational language to monitor inequalities by race, ethnicity or other characteristics besides sex, age, and persons with disabilities (currently no disaggregation is available in the database, however). Some human rights scholars argue: "Given the explicit target language, this cannot be considered an oversight [...] The reasons for this exclusion are likely to lie in the power of data: measurement of racial and ethnic inequalities may call attention to historic and ongoing injustices that those in power would prefer not to see highlighted in embarrassing data at the international level."[4] Our hope is that the new 'missing data' wedge, which was not included in the 2017 Doughnut, will help inspire others to fill this gap in global racial- and ethnic-equality monitoring, and that this could then, along with gender equality, serve as a more effective proxy for broader intersecting forms of discrimination.

Social cohesion
Social cohesion can be seen as a function of people's sense of belonging within a community, and of the strength of relationships between its members[1]. Social cohesion spans multiple SDGs but it is perhaps most closely related to the combined ambition to promote and enforce non-discriminatory laws and policies (SDG Target 16.b) and to reduce inequalities of outcome (SDG Target 10.3). Indicators monitoring progress on these targets are not currently available globally within the SDG Indicators database, however. We note that social cohesion is a new dimension in the Doughnut that replaces the previous 'social equity' dimension in the 2017 version, as described in the introduction to this social foundation section.

We include two indicators to monitor global social cohesion shortfalls in the Doughnut: (i) population stating that they are without someone to count on in times of trouble (World Happiness Report, WHR[46]), and (ii) population living in countries with a Palma ratio of 2 or more (the income share of the richest 10% of people relative to the poorest 40%; UNU-WIDER[47]).

Human beings hold a universal need to belong that motivates people to create and safeguard a sufficient quantity of meaningful, desired, and lasting interpersonal relationships[48]. An unmet need for belonging has been shown to mediate the relationship between levels of loneliness and psychological well-being[49]. National data on people's sense of belonging is not currently available for a large number of countries over our analysis period, however, so we collected data on self-reported social support as a reasonable proxy, notably given evidence showing clear links between the quality of social relationships and belonging[50].

National average social support data were collected from the *World Happiness Report 2024*[46], based on binary yes/no responses to the Gallup World Poll question: "If you were in trouble, do you have relatives or friends you can count on to help you whenever you need them, or not?" The national average of responses to this question can be interpreted as the proportion of population stating that they have someone to count on in times of trouble. We note that the results of this self-reported indicator should be interpreted with care given known cognitive biases, such as the availability heuristic that biases responses triggering emotionally charged memories, which may be more relevant when reducing the complexity of respondents' close relationships into a single yes/no question[51]. However, as the researchers leading the *World Happiness Report* project note, this is less likely an issue when

comparing national averages because individual differences in personality and life circumstances tend to average out at the national scale [46]. This indicator was also included in the 2017 Doughnut. Countries without observations before 2008 and with fewer than 10 observations in total were excluded, missing values were interpolated linearly to yield a balanced panel of 124 countries from 2005–2022, and population-weighted averages were calculated for the full sample and the subset of countries within each country cluster.

In terms of the strength of social relationships underpinning social cohesion, evidence suggests people living in more equal societies tend to be healthier, safer, better supported and more trusting compared to those in less equal societies[52]. Adopting policies that achieve greater equality – especially fiscal, wage, and social-protection policies – is the focus of SDG Target 10.4. The redistributive impact of pre- and post-fiscal policy on the Gini index – a common measure of income inequality – is provided in SDG Indicator 10.4.2[53], but data is not currently available globally. We used the Palma ratio to measure income inequalities, which is the ratio of the income of the top 10% to that of the bottom 40% within a nation. The Palma ratio is preferred over the Gini coefficient for our purposes because it has been shown to better reflect inequalities at the extremes of wealth and poverty[54]. This indicator was also included in the 2017 Doughnut and we set the same target at a Palma ratio of 2, i.e. when the richest 10% have double the annual income of the poorest 40%. National Palma ratio data were collected from the *World Income Inequality Database 2023*[47]. A balanced panel of 193 countries was collected over 2000–2022, and the population living in countries exceeding the Palma over-2 target was summed for the full sample and for the subset of countries within each country cluster. We note that this approach reports the share of global population in deprivation on the basis of country-level outcomes (rather than individual-level outcomes), which is sensitive to countries with large populations that cross the target.

Political voice
Democratic institutions, freedom of expression, freedom of association, and a free media have proven essential to enable social participation and personal autonomy[55], and to contest hegemonic discourses and divisive structures that concentrate power and opportunity into the hands of a wealthy few[56,57]. Ensuring responsive, inclusive, participatory, and representative decision-making at all levels is the focus of SDG Target 16.7. However, progress towards achieving this target is currently monitored in the SDG Indicators database using a set of more than 20 indicators with diverse country and time coverage. We include one widely used indicator to track global political voice shortfalls in the Doughnut: population living in countries governed by an autocratic regime (Varieties of Democracy (V-Dem) Institute[58]).

This indicator is a part of the V-Dem Institute's long-standing Regimes of the World classification, which uses expert evaluation to distinguish between four types of governance regimes: Closed Autocracy, Electoral Autocracy, Electoral Democracy and Liberal Democracy[58]. The classification is the result of country experts evaluating whether democratic characteristics are present or not, with inputs from more than 3,500 experts worldwide (V-Dem typically relies on 25 experts per country)[59]. A country is classified as an autocracy if the experts consider it lacks fundamental democratic components, such as freedom of expression and association, and free and fair elections. This indicator replaces Worldwide Governance Indicator's *Voice and Accountability Index* included in the 2017 Doughnut, which is a composite index that scores countries in relative terms (i.e. values are bounded by the best- and worst-performers observed empirically) that is not appropriate for our purposes of monitoring performance with respect to minimum social standards in absolute terms.

National governance regimes data were collected from V-Dem's *Democracy Report 2024*[58]. Countries with fewer than 10 observations in total were excluded, missing values were interpolated linearly to yield a balanced panel of 173 countries from 2000–2022, and the population living in countries classified as autocratic was summed for the full sample and for the subset of countries within each country cluster. . We note that this approach reports the share of global population in deprivation on the basis of country-level outcomes (rather than individual-level outcomes), which is sensitive to countries with large

populations that cross the target. For example, we observe a large worsening in this indicator in 2017 due to India being classified as a country governed by an autocratic regime in this year (see Extended Data Figure 5).

Peace & justice
Peaceful and just societies enable people to live free from fear, exploitation, and corruption. Promoting peaceful and inclusive societies and providing access to justice for all through accountable institutions are the foci of SDG Goal 16. We include two indicators to track peace and justice shortfalls in the Doughnut: (i) population living in countries with a homicide rate of 5 victims or more per 100,000 people (SDG 16.1.1[60]), and (ii) population stating that they perceive widespread corruption in government and business (World Happiness Report[46]).

Significantly reducing all forms of violence and related deaths everywhere is the focus of SDG Target 16.1. Intentional homicide is defined as the unlawful death inflicted upon a person with the intent to cause death or serious injury, which is reported across countries as the total count of victims divided by the population, expressed per 100,000 people[60]. This indicator is widely used at the international level as a direct indication of lack of security, although a composite indicator that also includes killings in war and conflict situations would be preferable.

The intentional homicide rate was also included in the 2017 Doughnut, although the minimum target has been revised here, down from 10 to 5 homicide victims per 100,000 people, based on the observation that countries with relatively high levels of violence, such as the Philippines and United States, are well within the 10 victims per 100,000 benchmark (7.5 and 5.4 victims per 100,000 people on average over the 2000-2022 period, respectively). National intentional homicide data were collected from the SDG Indicators database. Countries with fewer than five observations in the relatively sparse dataset were excluded, missing values were interpolated linearly to yield a balanced panel of 151 countries from 2000–2022, and the population living in countries exceeding the 5 victims per 100,000 people target was summed for the full sample and for the subset of countries within each country cluster. We note that this approach reports the share of global population in deprivation on the basis of country-level outcomes (rather than individual-level outcomes), which is sensitive to countries with large populations that cross the target.

Given the complex and elusive nature of corruption, self-reported evaluations can provide a straightforward means to capture perceptions of the extent to which public power is exercised for private gain, as well as perceptions of 'capture' of the state by elites and private interests. Although perceptions may be influenced by factors that are not directly linked to the prevalence of corruption, such as culture, propaganda, and values, they can provide a valuable proxy that combines direct experience of corruption with a sense of trust in institutions[61].

National average perceptions of corruption data were collected from the *World Happiness Report 2024*[46], based on the average of two questions with binary yes/no responses in the Gallup World Poll: "Is corruption widespread throughout the government or not?" and "Is corruption widespread within businesses or not?" The national average of responses to these questions can be interpreted as the proportion of population stating that they perceive widespread corruption in government and business. This indicator replaces Transparency International's *Corruption Perceptions Index* included in the 2017 Doughnut, which is a composite index that scores countries in relative terms (i.e. values are bounded by the best- and worst-performers observed empirically) that is not appropriate for our purposes of monitoring performance with respect to minimum social standards in absolute terms. Countries without observations before 2008 and with fewer than 10 observations in total were excluded, missing values were interpolated linearly to yield a balanced panel of 117 countries from 2005–2022, and population-weighted averages were calculated for the full sample and the subset of countries within each country cluster.

Supplementary Discussion 2 – The ecological ceiling: dimensions and indicators

We rename two of the Doughnut's ecological dimensions to reflect conceptual advances in planetary boundaries science since the previous update in 2017 (freshwater disruption and biodiversity breakdown). Freshwater disruption replaces 'freshwater withdrawals' in the 2017 Doughnut, as this boundary was strengthened to consider deviations in both streamflow (blue water) and soil moisture (green water)[62,63]. Biodiversity breakdown replaces 'biodiversity loss' in the 2017 Doughnut, as this boundary was revised to consider how excessive human appropriation of biomass and energy from photosynthetic organisms can undermine planetary health (alongside loss of species diversity). We also replace 'nitrogen and phosphorus loading' from the 2017 Doughnut with the term 'nutrient pollution' to be more accessible to a non-technical audience.

Drawing from the latest planetary boundaries update[64], we include three new indicators with relevant data to monitor ecological overshoot at the global scale compared to the previous version of the framework[65], namely green-water disruption, human appropriation of net primary productivity (HANPP), and interhemispheric aerosols. We note that our ambition to track ecological overshoot over time relies on several published data sources that are expressed in the same units as the planetary boundary indicators but are not explicitly referenced in the latest update (ozone layer[66], ocean acidification[67], nutrient pollution[68], and land conversion[69]).

In addition, we include six per capita environmental footprint proxy indicators[70] for our analysis of inequalities at the country-cluster scale, which are informed by planetary boundaries science, but are not directly comparable to the planetary boundaries. These downscaled per capita indicator-boundary pairs are related to four dimensions of the Doughnut's ecological ceiling (climate change, nutrient pollution, freshwater disruption, and biodiversity breakdown). The following sub-sections describe the revised set of dimensions and indicators included in the Doughnut's ecological ceiling, and Supplementary Table 2 provides an overview of the 19 ecological indicators included (13 at global scale; 6 at country-cluster scale), including country coverage, descriptions, and data sources.

Climate change
Fossil-fuel combustion, agriculture, and other human activities release greenhouse gases into the air, such as carbon dioxide, methane and nitrous oxide, which amplify Earth's natural greenhouse effect, causing an increase in temperatures, sea-level rise, and more intense droughts, floods and storms[71].

At the global scale, two indicator-boundary pairs from the planetary boundaries framework are included to track global climate change overshoot in the Doughnut: (i) atmospheric carbon dioxide concentration (at most 350 ppm CO_2)[72], and (ii) human-induced radiative forcing at the top of the atmosphere (at most 1 W/m^2)[72]. The atmospheric carbon dioxide concentration indicator-boundary pair is the same as in the 2017 Doughnut. The human-induced radiative forcing indicator-boundary pair is a new addition to the Doughnut compared to the 2017 version, as this was not included in the planetary boundary diagrams until the most recent update. Radiative forcing complements the CO_2 concentration indicator by monitoring the net change in the Earth's energy balance, which includes the combined warming and cooling effects of additional physical drivers of climate change, such as methane and nitrous oxide concentrations, aerosols, and surface albedo[71]. We note that both of the climate change boundaries are broadly consistent with 1ºC global heating beyond pre-industrial levels, which has already occurred, and they are substantially less risky than the 1.5ºC target set in the Paris Agreement on Climate Change[73]. We collected data for both indicators from *Indicators of Global Climate Change 2023*[72] over the 2000-2022 period, which is the same source as Richardson et al.[64]

At the country-cluster scale, one per capita indicator-boundary pair is included to track climate change overshoot in the 'downscaled' Doughnut: carbon footprint, i.e. CO_2 emissions per capita from fossil-fuel combustion embodied in the consumption of goods and services (at most 0.95 t CO_2 per capita per

year)[70]. National per capita carbon footprints were calculated as the arithmetic mean across expenditure decile values for each of the 168 countries with available data in the year 2017, and population-weighted averages were calculated for the subset of countries within each country cluster. See Tian et al.[70] for in-depth discussion of the caveats, uncertainties and limitations underpinning the methods and data.

Ocean acidification
The ocean has absorbed approximately 30% of the total carbon dioxide emitted by human activity, but this is increasing the acidity of seawater and reducing saturation levels of calcium carbonate minerals, which endangers marine ecosystems, notably by inhibiting organisms' shell and skeleton growth[74]. One indicator-boundary pair from the planetary boundaries framework is used to track global ocean acidification overshoot in the Doughnut: average saturation state of aragonite at the ocean surface (at least 80% of pre-industrial saturation state of 3.44 Ω_{arag})[67,75]. We collected data from the Japanese Meteorological Agency's *Global Ocean Carbon Uptake 2024* over the 2000–2022 period[75], which are updated on an ongoing annual basis, thus preferable for our time-series purposes to the estimates ending in the year 2010 from Jiang et al.[76] referenced by Richardson et al.[64]

Chemical pollution
Hazardous chemicals and synthetic compounds, such as organic pollutants and endocrine disruptors, can persist in the environment for a very long time, accumulating in the tissues of organisms, such as fish and mammals, with toxic effects including illness and death, reduced fertility, and genetic damage, thus endangering ecosystems on land and in the oceans[77]. One indicator-boundary pair is included to measure trends in global chemical pollution overshoot in the Doughnut: production of hazardous chemicals, millions of tonnes per year (at most 5% of the 1,200 Mt of total chemicals produced in year 2000)[77,78]. The hazardous chemicals indicator-boundary pair is a new addition to the Doughnut compared to the 2017 version, and it is related to but distinct from the qualitative approach included for the first time in the latest planetary boundaries update, based on the 'percentage of synthetic chemicals released to the environment without adequate safety testing'[64]. The main difference is that the quantitative metric included here measures the *production* of hazardous chemicals, as opposed to Richardson et al.'s[64] qualitative focus on *environmental release*.

Like Richardson et al.[64], we build on Persson et al.'s[79] landmark study that assesses seven control variables for the novel entity boundary along an impact pathway, from production to environmental release to Earth-system effect, based on their comprehensiveness, feasibility, and relevance. These authors find that *total chemical production* has high comprehensiveness, as harmful chemicals cannot be released to the environment if they are not produced in the first place. It also has high feasibility, as volumes are known by producers who can report these to regulators, thus data are easier to collect and aggregate than monitoring diffuse environmental releases. The relevance of total chemical production to Earth-system effects is more tenuous and difficult to assess, given the large number of chemicals with potential for myriad adverse impacts in the environment. That said, although links between chemical production and Earth-system effects may not be obvious, Persson et al.[79] note that total chemical production increases in relevance as a control variable based on the precautionary assumption of inevitable environmental release.

Persson et al.[79] also assess *total hazardous chemicals released into the environment* as a control variable, and find it has high relevance, since it tracks the scale of harmful chemicals with potential Earth-system effects, but low comprehensiveness and feasibility, largely because environmental releases are more dispersed and difficult to monitor. The authors do not explicitly assess *total hazardous chemicals production* as a control variable, but it can be inferred from their analysis that this variable likely has more relevance in comparison to total chemicals production, which includes both hazardous and non-hazardous chemicals. Similarly, a focus on hazardous chemicals production likely has more feasibility and comprehensiveness in comparison to hazardous chemicals released into the environment, where many small and dispersed sources are more difficult to monitor than large point sources.

Given the lack of a Holocene baseline for novel entities, the biomimicry aspiration to employ only life-friendly chemicals provides a compelling Nature-based ambition to create green chemistry that is conducive to life, rather than chemistry that is hazardous to health and environment[80–83]. The boundary for hazardous chemicals production is set at 5% of total chemical production of 1,200 Mt in the year 2000 - or 60 Mt per year. This boundary is larger than zero, recognising that the complex pathway from production to Earth-system impact is not necessarily one-to-one, although we caution that this value is highly uncertain as the literature is not mature in this area.

We acknowledge that the approach of defining the Doughnut's chemical pollution boundary is based on year 2000 values primarily due to data availability, as this is the earliest global total available from UNEP's landmark *Global Chemicals Outlook* series[77]. That said, given the weight of existing evidence showing that a safe boundary has already been transgressed[64,79], we believe it is useful to include the best available numerical proxy that we are aware of, as an input to further scientific debate. This approach coincides with efforts to define global maximum levels of total material resource use based on a return to year 2000 levels[84–86], which involves a similar challenge to defining a 'safe' level of a highly aggregated variable.

We collected global data on total chemical production from UNEP's *Global Chemicals Outlook II*[77], available for the years 2000 and 2017, interpolating linearly to create an annual series, and extrapolating linearly to extend the series to 2022. We could not identify a rigorous estimate of global hazardous chemicals production, so we collected data on the proportion of chemicals produced in Europe deemed hazardous to health, available 2004-2023 from Eurostat[78], which varies between 74.6% and 78.6% over the period, or 76.6% on average. We applied this average European share of hazardous chemicals to the total chemicals production estimates derived from UNEP to yield a rough approximation of the global share of hazardous chemicals production over the 2000-2022 period.

We note that other studies using different data sources and approaches find similarly high and concerning shares of potentially harmful chemical pollution in quantitative terms[79,87] . For example, Persson et al.[79] report that 80% of the ~23,000 chemicals registered under the EU's Registration, Evaluation, Authorisation and Restriction of Chemicals (REACH) regulation had been in public use for more than 10 years without yet having undergone a safety assessment. Wagner et al.[87] report that 92% of ~16,300 documented plastic chemicals can be classified as either hazardous (26%) or lacking hazard data (66%), with less than 1% classified as not hazardous. As such, our interim approach based on European production is likely to be a conservative underestimate of the share of hazardous chemicals in total chemical production, based on the observation that European chemical assessment and monitoring is arguably world-leading with comprehensive regulations in legal force since 2007[88].

Nutrient pollution

The widespread application of nitrogen (N) and phosphorus (P) fertilisers supports plant growth in agriculture, but most of these nutrients are washed into inland and coastal waters, causing algal blooms that can be toxic and/or that kill off other aquatic life by depleting the water's oxygen, which is essential for respiration[89,90].

At the global scale, two indicator-boundary pairs from the planetary boundaries framework are included to track global nutrient pollution in the Doughnut: (i) phosphorus applied to land as fertiliser (at most 6.2 Mt per year)[68], and (ii) reactive nitrogen applied to land as fertiliser (at most 62 Mt per year) [68]. Both indicator-boundary pairs are the same as in the 2017 Doughnut, although the previous 'nitrogen and phosphorus loading' dimension label has been replaced with 'nutrient pollution', as described in the introduction to this ecological ceiling section.

We collected global N and P data from Beusen et al.'s[68] landmark study that uses a comprehensive integrated assessment model with global nutrient module (IMAGE-GNM) to analyse soil N and P nutrient

budgets using historical observations from 1970-2015 and projecting future scenarios according to the Shared Socioeconomic Pathways (SSPs) from 2015-2050. For nitrogen, we extracted the global sum of nitrogen fixation and fertiliser/manure applied to arable land from 2000-2025, using the series with SSP2 assumptions for the 2015-2025 period, and interpolating linearly to create an annual 2000–2022 series from the 5-year data. For phosphorus, we extracted global phosphorus pentoxide inputs from fertilisers applied to arable land from 2000-2025, using the series with SSP2 assumptions for the 2015-2025 period, multiplying this series by the molar share of phosphorus (62/142), and interpolating linearly to create an annual 2000–2022 series from the 5-year data. We note that Beusen and colleagues[68,91] provide more recent estimates of N and P flows, including forward-looking projections, which are preferable for our time-series purposes to the estimates ending in 2013 from Lu and Tian[92] referenced by Richardson et al.[64]

At the country-cluster scale, two per capita indicator-boundary pairs are included to track nutrient pollution in the 'downscaled' Doughnut: (i) nitrogen footprint, i.e. nitrogen fertiliser per capita embodied in the consumption of goods and services (at most 8.5 kg N per capita per year), and (ii) phosphorus footprint, i.e. phosphorus fertiliser per capita embodied in the consumption of goods and services (at most 0.85 kg P per capita per year) [70]. National per capita nitrogen and phosphorus footprints were calculated as the arithmetic mean across expenditure decile values for each of the 168 countries with available data in the year 2017, and population-weighted averages were calculated for the subset of countries within each country cluster. See Tian et al.[70] for in-depth discussion of the caveats, uncertainties and limitations underpinning the methods and data.

Air pollution
Micro-particles, or aerosols, emitted into the air – such as dust, smoke, and other atmospheric pollutants – affect air quality and the health of living beings, and influence the climate through effects on cloud formation and on sunlight reaching Earth's surface[93]. One indicator-boundary pair from the planetary boundaries framework is included to track global air pollution overshoot in the Doughnut: asymmetry between Earth's hemispheres of sunlight reaching the surface, due to differences in atmospheric particle concentration (at most 0.1 inter-hemispheric difference in Aerosol Optical Depth), reported as a single value repeated over the 2000-2022 period due to a lack of time-series estimates[64,93]. The hemispheric asymmetry indicator-boundary pair is a new addition to the Doughnut compared to the 2017 version, which was included for the first time in the latest planetary boundaries update as a globally defined indicator for aerosol loading (unquantified in previous versions)[64]. Data were collected from Richardson et al.[64], originally reported as an average over the 1998-2020 period.

Freshwater disruption
The freshwater cycle provides water that is essential for life, but human activities are disrupting freshwater systems through excessive extraction, land conversion, and emissions of greenhouse gases and aerosols, which alter evaporation, soil moisture, precipitation, and runoff regimes[62,94].

At the global scale, two indicator-boundary pairs from the planetary boundaries framework are included to track global freshwater disruption in the Doughnut: (i) proportion of land area with human-induced disturbance of blue-water flow deviating from Holocene variability (at most 10.2%) over the 2000-2005 period[62], and (ii) proportion of land area with root-zone soil moisture deviating from Holocene variability (at most 11.1%) over the 2000-2014 period[62,63]. Both indicator-boundary pairs are new additions to the Doughnut compared to the 2017 version, as they replaced the singular focus on freshwater withdrawal volumes in the 2015 planetary boundaries framework, which had been criticised as being too blunt to account for changes in the complex freshwater cycle[94,95]. Freshwater disruption is a new dimension label in the Doughnut that replaces the 'freshwater withdrawals' dimension in the 2017 version, as described in the introduction to this ecological ceiling section.

Data on the proportions of land area with root-zone soil moisture and blue-water deviations during pre-industrial (1661-1860) and industrial periods (1861-2005) used in the 2023 planetary boundaries update are available from Porkka et al.[62] However, the 2005 end year is not well-suited for our time-series purposes. For soil moisture, we extended the Porkka et al.[62] series using data available from a related study by Wang-Erlandsson et al.[63] that estimates deviations up to the year 2014. We collected 2005-2014 data from Wang-Erlandsson et al., scaling the series to match and extend the 2005 values reported by Porkka et al. We are not aware of more recent estimates for blue-water deviations, so we collected the 2000-2005 series from Porkka et al.[62]. In both cases, we fill missing values by carrying the last observed value forward over the 2000-2022 period.

At the country-cluster scale, one per capita indicator-boundary pair is included to track freshwater disruption in the 'downscaled' Doughnut: blue water footprint, freshwater use per capita embodied in the consumption of goods and services (at most 384 m^3 H_2O per capita per year)[70]. National per capita blue water footprints were calculated as the arithmetic mean across expenditure decile values for each of the 168 countries with available data in the year 2017, and population-weighted averages were calculated for the subset of countries within each country cluster. See Tian et al.[70] for in-depth discussion of the caveats, uncertainties and limitations underpinning the methods and data.

Land conversion
Forests help maintain suitable conditions for life on Earth by housing biodiversity, storing carbon, and cycling water and nutrients, yet global forest area is shrinking by millions of hectares per year, with nearly three quarters of total deforestation due to agricultural expansion[69]. One indicator-boundary pair from the planetary boundaries framework is included to track global land conversion in the Doughnut: area of forested land as a proportion of forest-covered land prior to human alteration (at least 75% of 64 million square kilometres) over the 2000-2020 period[96]. This indicator-boundary pair is the same as in the 2017 Doughnut. We collected forest area data from FAO's *Global Forest Resources Assessment 2020*[96], interpolating linearly to create an annual series from the 10-year data, and extrapolating linearly to cover the 2000–2022 period. The resulting time-series value in 2019 is slightly higher than the 2019 value reported in the latest planetary boundaries update (40.6 versus 38.3 million km^2, respectively), so the FAO series was scaled to match the 2019 value reported by Richardson et al.[64]

Biodiversity breakdown
Biodiversity – the diversity within species, between species, and of ecosystems – is declining faster than at any time in human history[97].

At the global scale, two indicator-boundary pairs from the planetary boundaries framework are included to track global biodiversity breakdown in the Doughnut: (i) rate of species extinction (at most 10 extinctions per million species years), reported as a single value repeated over the 2000-2022 period due to a lack of time-series estimates[64,98], and (ii) human appropriation of net primary productivity in billions of tonnes of carbon per year (at most 10% of 55.9 Gt C) over the 2000-2020 period[64]. The extinction rate indicator-boundary pair is the same as in the 2017 Doughnut. The human appropriation of net primary productivity (HANPP) indicator-boundary pair is a new addition to the Doughnut compared to the 2017 version, which was included in the latest planetary boundaries update as a proxy to track the trophic energy flows (i.e. biomass) needed to maintain the functional integrity of ecosystems[64]. Data were collected for both indicator-boundary pairs from Richardson et al.[64] For the HANPP indicator, we interpolate linearly between the 2000 and 2020 values reported by Richardson et al., extrapolating to create an annual series over the 2000–2022 period. Biodiversity breakdown is a new dimension label in the Doughnut that replaces the previous 'biodiversity loss' dimension in the 2017 version, as described in the Main text.

At the country-cluster scale, two per capita indicator-boundary pairs are included to track biodiversity breakdown in the 'downscaled' Doughnut: (i) biodiversity footprint, mean species abundance loss per

hectare per capita embodied in the consumption of goods and services (at most 0.51 MSA-loss per hectare per capita per year), and (ii) HANPP footprint, human appropriation of net primary productivity per capita embodied in the consumption of goods and services (at most 0.74 t C per capita per year) [70]. National per capita species-loss and HANPP footprints were calculated as the arithmetic mean across expenditure decile values for each of the 168 countries with available data in the year 2017, and population-weighted averages were calculated for the subset of countries within each country cluster. See Tian et al.[70] for in-depth discussion of the caveats, uncertainties and limitations underpinning the methods and data.

Ozone layer depletion

Ozone in the stratosphere protects the Earth from the sun's biologically harmful ultraviolet radiation, but the emission of long-lived ozone-depleting substances, such as chlorofluorocarbons (CFCs), throughout most of the 20th century led to a notable decline in the abundance of stratospheric ozone[99]. This declining trend was largely halted by 1995 due to successful global regulations on the use of ozone-depleting substances, and the global ozone layer is now slowly recovering and expected to reach pre-1980 abundance around the middle of the 21st century (assuming continued compliance)[66,99]. One indicator-boundary pair from the planetary boundaries framework is included to track global ozone layer depletion in the Doughnut: concentration of ozone in the stratosphere (at most 5% decrease with respect to 1964-80 value of 290 Dobson Units) over the 2000-2021 period[66]. The ozone layer depletion indicator-boundary pair is the same as in the 2017 Doughnut. We collected data from Weber et al.'s[66] landmark analysis that merges major global ozone datasets over the 1964-2021 period, which informs the World Meteorological Organisation's ongoing *Scientific Assessment of Ozone Depletion*[99] series (updated at least every four years). This source was preferred for our time-series purposes to NASA's Aura satellite data[100] beginning in 2012 referenced by Richardson et al.[64]

Supplementary Tables

Supplementary Table 1. Data sources for the social foundation and its indicators of shortfall

Dimension	Sample	Indicator	Period	Source
Food	*N* = 166	Population undernourished	2000-2022	SDG 2.1.1[7]
	N = 126	Population with moderate to severe food insecurity	2014-2022	SDG 2.1.2[8]
Health	*N* = 191	Population living in countries with under-five mortality rate exceeding 25 per 1,000 live births	2000-2022	SDG 3.2.1[15]
	N = 190	Population living in countries lacking high coverage of essential health services (Universal Health Coverage Index score less than 60)	2000-2022	SDG 3.8.1[16]
Education	*N* = 127	Adult population (aged 15+) who are illiterate	2000-2022	UNESCO[18]
	N = 116	Young adult population (aged 21–23) with incomplete upper secondary education	2000-2022	SDG 4.1.2[19]
Income & work	*N* = 112	Population living below the societal poverty line, set at half their country's median household income or at least $15 a day	2000-2022	World Bank[31]
	N = 119	Population of young people (aged 15–24) not in employment, education, or training	2005-2022	SDG 8.6.1[24]
Water & sanitation	*N* = 124	Population lacking access to safely managed drinking water	2000-2022	SDG 6.1.1[33]
	N = 131	Population lacking access to safely managed sanitation	2000-2022	SDG 6.2.1[34]
Energy	*N* = 193	Population lacking access to electricity	2000-2022	SDG 7.1.1[35]
	N = 187	Population lacking access to clean fuels and technologies for cooking, heating, and lighting	2000-2022	SDG 7.1.2[36]
Connectivity	*N* = 177	Urban population lacking convenient access to public transport	2020	SDG 11.2.1[42]
	N = 190	Population not accessing the Internet	2000-2022	SDG 17.8.1[43]
Housing	*N* = 170	Urban population living in slums or informal settlements	2000-2022	SDG 11.1.1[44]
Equality	*N* = 155	Population-weighted score on the Gender Inequality Index (gap between women and men in terms of reproductive health, empowerment, and employment)	2000-2022	UNDP[6]
	--	Racial inequality (no global indicator currently tracks racial and ethnic equality gaps for social outcomes)	--	--
Social cohesion	*N* = 124	Population stating that they are without someone to count on in times of trouble	2005-2022	Gallup / WHR[46]
	N = 193	Population living in countries with a Palma ratio of 2 or more (the income share of the richest 10% of people relative to the poorest 40%)	2000-2022	UNU WIDER[47]
Political voice	*N* = 124	Population living in countries governed by an autocratic regime	2000-2022	V-DEM[58]
Peace & justice	*N* = 151	Population living in countries with a homicide rate of 5 or more per 100,000	2000-2022	SDG 16.1.1[60]
	N = 117	Population stating that they perceive widespread corruption in government and business	2005-2022	Gallup / WHR[46]

Supplementary Table 2. Data sources for the ecological ceiling and its indicators of overshoot

Dimension	Sample	Indicator (and planetary boundary)	Baseline	Period	Source
Climate change	Global	Atmospheric carbon dioxide concentration, parts per million (at most 350 ppm CO_2)	280 ppm CO_2	2000-2022	Forster et al.[72]
	Global	Human-induced radiative forcing at the top of the atmosphere, Watt per square metre (at most 1 W/m^2)	0 W/m^2	2000-2022	Forster et al.[72]
	N = 168	Carbon footprint, CO_2 emissions per capita from fossil fuel combustion embodied in the consumption of goods and services (at most 0.95 t CO_2 per capita per year)	0 t CO_2	2017	Tian et al.[70]
Ocean acidification	Global	Average saturation state of aragonite at the ocean surface (at least 80% of pre-industrial saturation state)	3.44 Ω_{arag}	2000-2022	Iida et al.[67]; JMA[75]
Chemical pollution	Global	Production of hazardous chemicals, millions of tonnes per year (at most 5% of the 1,200 Mt of total chemicals produced in year 2000)	0 Mt	2000-2017	UNEP[77]; Eurostat[78]
Nutrient pollution	Global	Phosphorus applied to land as fertiliser, millions of tonnes (at most 6.2 Mt P per year)	0 Mt P per year	2000-2022	Beusen et al.[68]
	Global	Reactive nitrogen applied to land as fertiliser, millions of tonnes (at most 62 Mt per year)	0 Mt N per year	2000-2022	Beusen et al.[68]
	N = 168	Phosphorus footprint, phosphorus fertiliser per capita embodied in the consumption of goods and services (at most 0.85 kg P per capita per year)	0 kg P per year	2017	Tian et al.[70]
	N = 168	Nitrogen footprint, nitrogen fertiliser per capita embodied in the consumption of goods and services (at most 8.5 kg N per capita per year)	0 kg N per year	2017	Tian et al.[70]
Air pollution	Global	Asymmetry between Earth's hemispheres of sunlight reaching the surface, due to differences in atmospheric particle concentration (at most 0.1 inter-hemispheric difference in Aerosol Optical Depth)	0.03	Most recent	Richardson et al.[64]
Freshwater disruption	Global	Proportion of land area with human-induced disturbance of blue-water flow deviating from Holocene variability (at most 10.2%)	0%	2000-2005	Porkka et al.[62]
	Global	Proportion of land area with root-zone soil moisture deviating from Holocene variability (at most 11.1%)	0%	2000-2014	Porkka et al.[62]; Wang-Erlandsson et al.[63]
	N = 168	Blue water footprint, freshwater use per capita embodied in the consumption of goods and services (at most 384 m^3 H_2O per capita per year)	0 m^3 H_2O	2017	Tian et al.[70]
Land conversion	Global	Area of forested land as a proportion of forest-covered land prior to human alteration (at least 75% of pre-industrial forest-covered land)	64 Mkm^2	2000-2020	FAO[96]; Richardson et al.[64]
Biodiversity breakdown	Global	Rate of species extinction per million species years (at most 10 E/MSY)	1 E/MSY	Most recent	Richardson et al.[64]
	Global	Human appropriation of net primary productivity, billions of tonnes of carbon per year (at most 10% of pre-industrial Holocene net primary productivity)	55.9 Gt C	2000-2020	Richardson et al.[64]
	N = 168	Biodiversity footprint, mean species abundance loss per hectare per capita embodied in the consumption of goods and services (at most 0.51 MSA-loss per hectare per capita per year)	0 MSA-loss per ha	2017	Tian et al.[70]
	N = 168	HANPP footprint, human appropriation of net primary productivity per capita embodied in the consumption of goods and services (at most 0.74 t C per capita per year)	0 t C	2017	Tian et al.[70]
Ozone layer depletion	Global	Concentration of ozone in the stratosphere, Dobson Units (at most 5% decrease with respect to 1964-80 value)	290 DU	2000-2021	Weber et al.[66]

Supplementary References

1. Manca, A. R. Social Cohesion. in *Encyclopedia of Quality of Life and Well-Being Research* (ed. Michalos, A. C.) 6026–6028 (Springer Netherlands, Dordrecht, 2014). doi:10.1007/978-94-007-0753-5_2739.
2. UN DESA. SDG Indicators Database. Statistics Division (2024).
3. UN Human Rights Council. *2030 Agenda for Sustainable Development, the Sustainable Development Goals and the Fight Against Racial Discrimination: Report of the Special Rapporteur on Contemporary Forms of Racism, Racial Discrimination, Xenophobia and Related Intolerance, E. Tendayi Achiume*. https://www.ohchr.org/en/press-releases/2022/07/special-rapporteur-racism-human-rights-council-2030-agenda-characterised (2022).
4. Winkler, I. T. & Satterthwaite, M. L. Leaving no one behind? Persistent inequalities in the SDGs. *Int. J. Hum. Rights* **21**, 1073–1097 (2017).
5. UN Population Division. *World Population Prospects 2022*. https://population.un.org/wpp/ (2022).
6. UNDP. *Human Development Report 2023-2024 - Breaking the Gridlock: Reimagining Cooperation in a Polarized World*. https://hdr.undp.org/content/human-development-report-2023-24 (2024).
7. UNSDG. Indicator 2.1.1: Prevalence of undernourishment (%). SDG Indicators Database https://unstats.un.org/sdgs/dataportal/SDMXMetadataPage?2.1.1-SN_ITK_DEFC (2023).
8. UNSDG. Indicator 2.1.2: Prevalence of moderate or severe food insecurity in the population (%). SDG Indicators Database https://unstats.un.org/sdgs/dataportal/SDMXMetadataPage?2.1.2-AG_PRD_FIESMS (2023).
9. Ritchie, H. How is food insecurity measured? *Our World Data* (2023).
10. IFAD. *The Land Tenure Security Advantage: A Catalytic Asset for Sustainable and Inclusive Rural Transformation*. https://www.ifad.org/documents/38714170/42014188/landtenure_security_advantage.pdf/319aa9a0-6b5e-a9b6-8f0d-21c391e3dc57?t=1596463568000 (2020).
11. UNSDG. SDG Indicator 1.4.2: Proportion of people with secure tenure rights to land out of total adult population (%). SDG Indicators Database https://unstats.un.org/sdgs/dataportal/SDMXMetadataPage?1.4.2-SP_LGL_LNDSTR (2024).
12. de Haen, H., Klasen, S. & Qaim, M. What do we really know? Metrics for food insecurity and undernutrition. *Food Policy* **36**, 760–769 (2011).
13. Ballard, T. J., Kepple, A. W. & Cafiero, C. *Food Insecurity Experience Scale: Development of A Global Standard for Monitoring Hunger Worldwide*. https://www.fao.org/3/as583e/as583e.pdf (2013).
14. Lain, J., Tandon, S. & Vishwanath, T. How Much Does the Food Insecurity Experience Scale Overlap with Poor Food Consumption and Monetary Poverty? Evidence from West Africa. *World Bank Econ. Rev.* **38**, 422–442 (2024).
15. UNSDG. Indicator 3.2.1: Under-5 mortality rate. SDG Indicators Database https://unstats.un.org/sdgs/dataportal/SDMXMetadataPage?3.2.1-SH_DYN_MORT (2023).
16. UNSDG. SDG Indicator 3.8.1: Universal health coverage (UHC) service coverage index. SDG Indicators Database https://unstats.un.org/sdgs/dataportal/SDMXMetadataPage?3.8.1-SH_ACS_UNHC (2023).
17. WHO & World Bank. *Tracking Universal Health Coverage: 2023 Global Monitoring Report*. (2023).
18. UNESCO. Literacy rate, adult total (% of people ages 15 and above). (2023).
19. UNSDG. Indicator 4.1.2: Completion rate (upper secondary education). SDG Indicators Database https://unstats.un.org/sdgs/dataportal/SDMXMetadataPage?4.1.2-SE_TOT_CPLR (2023).
20. UNSDG. SDG Indicator 4.6.1: Proportion of population achieving at least a fixed level of proficiency in functional literacy and numeracy skills. SDG Indicators Database https://unstats.un.org/sdgs/dataportal/SDMXMetadataPage?4.6.1-SE_ADT_FUNS (2023).

21. World Bank. World Development Indicators. https://databank.worldbank.org (2023).

22. Cohen, J. E. Make secondary education universal. *Nature* **456**, 572–573 (2008).

23. World Bank. *Poverty and Shared Prosperity 2018: Piecing Together the Poverty Puzzle*. https://openknowledge.worldbank.org/server/api/core/bitstreams/34b63a7c-7c89-5474-b9d3-f4f35085fe83/content (2018).

24. UNSDG. Indicator 8.6.1: Proportion of youth (aged 15-24 years) not in education, employment or training (%). SDG Indicators Database https://unstats.un.org/sdgs/dataportal/SDMXMetadataPage?8.6.1-SL_TLF_NEET (2023).

25. World Bank. *Monitoring Global Poverty: Report of the Commission on Global Poverty*. https://doi.org/10.1596/978-1-4648-0961-3 (2017) doi:10.1596/978-1-4648-1602-4.

26. Moatsos, M. Global Absolute Poverty: Behind the Veil of Dollars. *J. Glob. Dev.* **7**, 20160033 (2017).

27. Sullivan, D., Moatsos, M. & Hickel, J. Capitalist reforms and extreme poverty in China: unprecedented progress or income deflation? *New Polit. Econ.* **0**, 1–21 (2023).

28. Jolliffe, D., Mahler, D. G., Lakner, C., Atamanov, A. & Tetteh-Baah, S. K. *Assessing the Impact of the 2017 PPPs on the International Poverty Line and Global Poverty*. https://documents1.worldbank.org/curated/en/353811645450974574/pdf/Assessing-the-Impact-of-the-2017-PPPs-on-the-International-Poverty-Line-and-Global-Poverty.pdf (2022).

29. Edward, P. & Sumner, A. Global poverty and inequality: are the revised estimates open to an alternative interpretation? *Third World Q.* **39**, 487–509 (2018).

30. Fanning, A. L., O'Neill, D. W., Hickel, J. & Roux, N. The social shortfall and ecological overshoot of nations. *Nat. Sustain.* **5**, 26–36 (2022).

31. World Bank. Poverty & Inequality Indicators. Poverty and Inequality Platform https://pip.worldbank.org/poverty-calculator (2023).

32. Berten, J. Producing decent work indicators: contested numbers at the ILO. *Policy Soc.* **41**, 458–470 (2022).

33. UNSDG. Indicator 6.1.1: Proportion of population using safely managed drinking water services (%). SDG Indicators Database https://unstats.un.org/sdgs/dataportal/SDMXMetadataPage?6.1.1-SH_H2O_SAFE (2023).

34. UNSDG. Indicator 6.2.1: Proportion of population using safely managed sanitation services (%). SDG Indicators Database https://unstats.un.org/sdgs/dataportal/SDMXMetadataPage?6.2.1-SH_SAN_SAFE (2023).

35. UNSDG. Indicator 7.1.1: Proportion of population with access to electricity (%). SDG Indicators Database https://unstats.un.org/sdgs/dataportal/SDMXMetadataPage?7.1.1-EG_ACS_ELEC (2023).

36. UNSDG. Indicator 7.1.2: Proportion of population with primary reliance on clean fuels and technology (%). SDG Indicators Database https://unstats.un.org/sdgs/dataportal/SDMXMetadataPage?7.1.2-EG_EGY_CLEAN (2023).

37. Bhatia, M. & Angelou, N. *Beyond Connections: Energy Access Redefined*. https://mtfenergyaccess.esmap.org/data/files/download-documents/full_report_beyond_connection.pdf (2015).

38. Ayaburi, J., Bazilian, M., Kincer, J. & Moss, T. Measuring "Reasonably Reliable" access to electricity services. *Electr. J.* **33**, 106828 (2020).

39. Kikstra, J. S., Mastrucci, A., Min, J., Riahi, K. & Rao, N. D. Decent living gaps and energy needs around the world. *Environ. Res. Lett.* **16**, 095006 (2021).

40. Min, B. *et al.* Lost in the dark: A survey of energy poverty from space. *Joule* **8**, 1982–1998 (2024).

41. *WHO Indoor Air Quality Guidelines: Household Fuel Combustion*. (World Health Organization, Geneva, 2014).

42. UNSDG. Indicator 11.2.1: Proportion of population that has convenient access to public transport (%). SDG Indicators Database https://unstats.un.org/sdgs/dataportal/SDMXMetadataPage?11.2.1-SP_TRN_PUBL (2023).

43. UNSDG. Indicator 17.8.1: Proportion of individuals using the internet (%). SDG Indicators Database https://unstats.un.org/sdgs/dataportal/SDMXMetadataPage?17.8.1-IT_USE_ii99 (2023).
44. UNSDG. Indicator 11.1.1: Proportion of urban population living in slums, informal settlements or inadequate housing. SDG Indicators Database https://unstats.un.org/sdgs/dataportal/SDMXMetadataPage?11.1.1-EN_LND_SLUM (2023).
45. UNSDG. SDG Indicator 10.2.1: Proportion of people living below 50 percent of median income by sex, age and persons with disabilities. SDG Indicators Database https://unstats.un.org/sdgs/dataportal/SDMXMetadataPage?10.2.1-SI_POV_50MI (2023).
46. Helliwell, J. *et al. World Happiness Report 2024*. https://worldhappiness.report/ed/2024/happiness-and-age-summary (2024) doi:10.18724/WHR-KK3M-B586.
47. UNU WIDER. World Income Inequality Database - WIID. World Income Inequality Database http://www.wider.unu.edu/database/world-income-inequality-database-wiid (2023).
48. Baumeister, R. F. & Leary, M. R. The need to belong: desire for interpersonal attachments as a fundamental human motivation. *Psychol. Bull.* **117**, 497–529 (1995).
49. Mellor, D., Stokes, M., Firth, L., Hayashi, Y. & Cummins, R. Need for belonging, relationship satisfaction, loneliness, and life satisfaction. *Personal. Individ. Differ.* **45**, 213–218 (2008).
50. Allen, K.-A., Kern, M. L., Rozek, C. S., McInerney, D. M. & Slavich, G. M. Belonging: a review of conceptual issues, an integrative framework, and directions for future research. *Aust. J. Psychol.* **73**, 87–102 (2021).
51. Tversky, A. & Kahneman, D. Judgment under Uncertainty: Heuristics and Biases. *Science* **185**, 1124–1131 (1974).
52. Wilkinson, R. & Pickett, K. *The Spirit Level: Why Greater Equality Makes Societies Stronger*. (Bloomsbury Publishing, New York, NY, 2011).
53. UNSDG. SDG Indicator 10.4.2: Redistributive impact of fiscal policy, Gini index (%). SDG Indicators Database https://unstats.un.org/sdgs/dataportal/SDMXMetadataPage?10.4.2-SI_DST_FISP (2024).
54. Cobham, A., Schlögl, L. & Sumner, A. Inequality and the Tails: the Palma Proposition and Ratio. *Glob. Policy* **7**, 25–36 (2016).
55. Doyal, L. & Gough, I. *A Theory of Human Need*. (Red Globe Press, Basingstoke, Hampshire, 1991).
56. Lamb, W. F. *et al.* Discourses of climate delay. *Glob. Sustain.* **3**, (2020).
57. Raworth, K. Why it's time for Doughnut Economics. *IPPR Progress. Rev.* **24**, 216–222 (2017).
58. Nord, M. *et al. Democracy Report 2024: Democracy Winning and Losing at the Ballot*. https://v-dem.net/publications/democracy-reports/ (2024).
59. Coppedge, M. *et al. V-Dem Methodology V14*. https://www.v-dem.net/documents/39/v-dem_methodology_v14.pdf (2024).
60. UNSDG. Indicator 16.1.1: Number of victims of intentional homicide (victims per 100,000 population). SDG Indicators Database https://unstats.un.org/sdgs/dataportal/SDMXMetadataPage?16.1.1-VC_IHR_PSRC (2023).
61. UNODC. *Statistical Framework to Measure Corruption*. https://www.unodc.org/documents/data-and-analysis/statistics/corruption/UNODC_Statistical_Framework_to_measure_corruption.pdf (2023).
62. Porkka, M. *et al.* Notable shifts beyond pre-industrial streamflow and soil moisture conditions transgress the planetary boundary for freshwater change. *Nat. Water* **2**, 262–273 (2024).
63. Wang-Erlandsson, L. *et al.* A planetary boundary for green water. *Nat. Rev. Earth Environ.* **3**, 380–392 (2022).
64. Richardson, K. *et al.* Earth beyond six of nine planetary boundaries. *Sci. Adv.* **9**, eadh2458 (2023).
65. Steffen, W. *et al.* Planetary boundaries: Guiding human development on a changing planet. *Science* **347**, (2015).

66. Weber, M. *et al.* Global total ozone recovery trends attributed to ozone-depleting substance (ODS) changes derived from five merged ozone datasets. *Atmospheric Chem. Phys.* **22**, 6843–6859 (2022).

67. Iida, Y., Takatani, Y., Kojima, A. & Ishii, M. Global trends of ocean CO2 sink and ocean acidification: an observation-based reconstruction of surface ocean inorganic carbon variables. *J. Oceanogr.* **77**, 323–358 (2021).

68. Beusen, A. H. W. *et al.* Exploring river nitrogen and phosphorus loading and export to global coastal waters in the Shared Socio-economic pathways. *Glob. Environ. Change* **72**, 102426 (2022).

69. FAO. *Global Forest Resources Assessment 2020*. (United Nations Food and Agriculture Organisation, Rome, 2020).

70. Tian, P. *et al.* Keeping the global consumption within the planetary boundaries. *Nature* **635**, 625–630 (2024).

71. Hoesung, L. *et al. Synthesis Report of the IPCC Sixth Assessment Report (AR6): Summary for Policymakers*. (2023).

72. Forster, P. M. *et al.* Indicators of Global Climate Change 2023: annual update of key indicators of the state of the climate system and human influence. *Earth Syst. Sci. Data* **16**, 2625–2658 (2024).

73. Rockström, J. *et al.* Safe and just Earth system boundaries. *Nature* **619**, 102–111 (2023).

74. Doney, S. C., Busch, D. S., Cooley, S. R. & Kroeker, K. J. The Impacts of Ocean Acidification on Marine Ecosystems and Reliant Human Communities. *Annu. Rev. Environ. Resour.* **45**, 83–112 (2020).

75. Japanese Meterological Agency. Carbon dioxide mapping data (JMA Ocean CO2 Map). Global Ocean Carbon Dioxide Uptake https://www.data.jma.go.jp/kaiyou/english/co2_flux/co2_flux_data_en.html (2024).

76. Jiang, L.-Q. *et al.* Climatological distribution of aragonite saturation state in the global oceans. *Glob. Biogeochem. Cycles* **29**, 1656–1673 (2015).

77. UNEP. *Global Chemicals Outlook II - From Legacies to Innovative Solutions: Implementing the 2030 Agenda for Sustainable Development*. https://www.unep.org/resources/report/global-chemicals-outlook-ii-legacies-innovative-solutions (2019).

78. Eurostat. Chemicals production and consumption statistics. Statistics Explained https://ec.europa.eu/eurostat/statistics-explained/index.php?title=Chemicals_production_and_consumption_statistics (2024).

79. Persson, L. *et al.* Outside the Safe Operating Space of the Planetary Boundary for Novel Entities. *Environ. Sci. Technol.* (2022) doi:10.1021/acs.est.1c04158.

80. Zimmerman, J. B., Anastas, P. T., Erythropel, H. C. & Leitner, W. Designing for a green chemistry future. *Science* **367**, 397–400 (2020).

81. Lane, M. K. M. *et al.* Green chemistry as just chemistry. *Nat. Sustain.* **6**, 502–512 (2023).

82. Hayes, S., Desha, C. & Gibbs, M. Findings of Case-Study Analysis: System-Level Biomimicry in Built-Environment Design. *Biomimetics* **4**, 73 (2019).

83. Caldeira, C. *et al. Safe and Sustainable by Design Chemicals and Materials: Review of Safety and Sustainability Dimensions, Aspects, Methods, Indicators, and Tools*. (Joint Research Centre, Luxembourg, 2022).

84. Bringezu, S. Possible Target Corridor for Sustainable Use of Global Material Resources. *Resources* **4**, 25–54 (2015).

85. O'Neill, D. W., Fanning, A. L., Lamb, W. F. & Steinberger, J. K. A good life for all within planetary boundaries. *Nat. Sustain.* **1**, 88–95 (2018).

86. Hickel, J., O'Neill, D. W., Fanning, A. L. & Zoomkawala, H. National responsibility for ecological breakdown: a fair-shares assessment of resource use, 1970–2017. *Lancet Planet. Health* **6**, e342–e349 (2022).

87. Wagner, M. *et al. State-of-the-Science on Plastic Chemicals - Identifying and Addressing Chemicals and Polymers of Concern*. https://plastchem-project.org/ (2024).

88. EEA and ECHA. *EU Indicator Framework for Chemicals*. https://www.eea.europa.eu/publications/eu-indicator-framework-for-chemicals (2024).

89. Diaz, R. J. & Rosenberg, R. Spreading Dead Zones and Consequences for Marine Ecosystems. *Science* **321**, 926–929 (2008).

90. UNEP. *Global Environment Outlook 6 Summary for Policymakers*. http://www.unep.org/resources/assessment/global-environment-outlook-6-summary-policymakers (2019).

91. Beusen, A. H. W., Bouwman, A. F., Van Beek, L. P. H., Mogollón, J. M. & Middelburg, J. J. Global riverine N and P transport to ocean increased during the 20th century despite increased retention along the aquatic continuum. *Biogeosciences* **13**, 2441–2451 (2016).

92. Lu, C. & Tian, H. Global nitrogen and phosphorus fertilizer use for agriculture production in the past half century: shifted hot spots and nutrient imbalance. *Earth Syst. Sci. Data* **9**, 181–192 (2017).

93. Vogel, A. *et al.* Uncertainty in Aerosol Optical Depth From Modern Aerosol-Climate Models, Reanalyses, and Satellite Products. *J. Geophys. Res. Atmospheres* **127**, e2021JD035483 (2022).

94. Gleeson, T. *et al.* The Water Planetary Boundary: Interrogation and Revision. *One Earth* **2**, 223–234 (2020).

95. Heistermann, M. HESS Opinions: A planetary boundary on freshwater use is misleading. *Hydrol. Earth Syst. Sci.* **21**, 3455–3461 (2017).

96. FAO. Global Forest Resources Assessment 2020: Forest Area (1990-2020). Forest Resources Assessment Data https://fra-data.fao.org/assessments/fra/2020 (2020).

97. IPBES. *Summary for Policymakers of the Global Assessment Report on Biodiversity and Ecosystem Services*. https://zenodo.org/records/3553579 (2019) doi:10.5281/zenodo.3553579.

98. Rounsevell, M. D. A. *et al.* A biodiversity target based on species extinctions. *Science* **368**, 1193–1195 (2020).

99. Salawitch, R. *et al. Twenty Questions and Answers About the Ozone Layer: 2022 Update*. https://ozone.unep.org/science/assessment/sap (2023).

100. NASA Earth Observation. AURA Ozone Data. NASA https://neo.gsfc.nasa.gov/archive/csv/AURA_OZONE_M/ (2024).
